# Tracking Immunoglobulin Repertoire and Transcriptomic Changes in Germinal Center B Cells by Single-Cell Analysis

**DOI:** 10.3389/fimmu.2021.818758

**Published:** 2022-01-12

**Authors:** Clarissa Corinaldesi, Antony B. Holmes, Qiong Shen, Eli Grunstein, Laura Pasqualucci, Riccardo Dalla-Favera, Katia Basso

**Affiliations:** ^1^ Institute for Cancer Genetics, Columbia University, New York, NY, United States; ^2^ Department of Otolaringology Head and Neck Surgery, Columbia University, New York, NY, United States; ^3^ Department of Pathology and Cell Biology, Columbia University, New York, NY, United States; ^4^ The Herbert Irving Comprehensive Cancer Center, Columbia University, New York, NY, United States; ^5^ Department of Microbiology and Immunology, Columbia University, New York, NY, United States; ^6^ Department of Genetics and Development, Columbia University, New York, NY, United States

**Keywords:** germinal center, single-cell analysis, gene expression, memory B cells, plasma cells, B cell receptor

## Abstract

In response to T-cell-dependent antigens, mature B cells in the secondary lymphoid organs are stimulated to form germinal centers (GCs), which are histological structures deputed to antibody affinity maturation, a process associated with immunoglobulin gene editing by somatic hypermutation (SHM) and class switch recombination (CSR). GC B cells are heterogeneous and transition across multiple stages before being eliminated by apoptosis or committing to post-GC differentiation as memory B cells or plasma cells. In order to explore the dynamics of SHM and CSR during the GC reaction, we identified GC subpopulations by single-cell (sc) transcriptomics and analyzed the load of immunoglobulin variable (V) region mutations as well as the isotype class distribution in each subpopulation. The results showed that the large majority of GC B cells display a quantitatively similar mutational load in the V regions and analogous IGH isotype class distribution, except for the precursors of memory B cells (PreM) and plasma cells (PBL). PreM showed a bimodal pattern with about half of the cells displaying high V region germline identity and enrichment for unswitched IGH, while the rest of the cells carried a mutational load similar to the bulk of GC B cells and showed a switched isotype. PBL displayed a bias toward expression of IGHG and higher V region germline identity compared to the bulk of GC B cells. Genes implicated in SHM and CSR were significantly induced in specific GC subpopulations, consistent with the occurrence of SHM in dark zone cells and suggesting that CSR can occur within the GC.

## Introduction

Germinal centers (GCs) are histological structures, which form in the secondary lymphoid organs in response to antigenic challenge. They represent the site of antibody affinity maturation, based on the acquisition of mutations in the immunoglobulin variable regions followed by selection based on the affinity for the antigen (1). The GC has been traditionally divided into two histological and functionally distinct compartments: the dark zone (DZ), representing the site where cells proliferate and immunoglobulin somatic hypermutation (SHM) occurs, and the light zone (LZ), where affinity-based selection takes place (2–4). More recent insights in GC biology, mostly based on single cell (sc)-RNAseq analyses of mouse and human cells, clearly showed that the GC reaction is better explained by a continuum of cell states between the DZ and the LZ (5, 6). Indeed, about one third of GC B cells display a transcriptomic and phenotypic profile that is intermediate between DZ and LZ features (7–10). In addition, a small subset of cells in the GC are primed to become memory B cells or plasma cells. These precursors of post-GC effector B cells are clearly distinct and recent sc-RNAseq studies have further expanded their transcriptome characterization (7, 10–13).

Mature B cells rely on their B cell receptors (BCR) for survival and for affinity selection prior to differentiation into memory B cells and plasma cells (14–16). Affinity maturation starts with the introduction of mutations in the variable regions of the immunoglobulin genes by SHM, a mechanism occurring in DZ GC B cells (17, 18). GC B cells appear to be selected based on the newly acquired affinity, with high-affinity cells preferentially differentiating into plasma cells and low-affinity cells into memory B cells (19, 20). The process of affinity maturation relies on multiple rounds of SHM in the DZ followed by selection in the LZ, with cells recirculating between the two compartments (4, 21, 22).

Antibody effector functions are instead determined by the isotype class that is defined by the expressed IGH constant region. The expression of the IGHD and IGHM constant chains can be replaced by different constant regions through the mechanism of class switch recombination (CSR) (23). The original notion that CSR occurs in the GC LZ has been challenged by several observations in mice suggesting that B cells undergo CSR before entering the GC reaction (24–26). More recent data in human cells were consistent with CSR occurring before GC formation, although not excluding that CSR may happen also in the GC (10).

We have recently investigated the transcriptome of GC B cells by sc-RNAseq profiling and identified a number of functionally linked subpopulations (7). We have now extended this analysis to track the consequences of SHM, CSR and affinity selection in each of the GC subpopulations by paired transcriptomic and antibody repertoire analyses at the single cell level. The results highlight a remarkably homogeneous distribution of mutational patterns across multiple DZ, LZ and intermediate cells, with the exception of cells committed to memory or plasma cell differentiation.

## Methods

### Cell Isolation

Palatine tonsils were obtained at the Children’s Hospital of Columbia-Presbyterian Medical Center as residual material from three anonymous patients who had undergone elective tonsillectomy due to chronic tonsillitis in compliance with Regulatory Guideline 45 CFR 46.101 (b) (4) for Exempt Human Research Subjects of the U.S. Department of Health and Human Services and according to protocols approved by the Columbia University Institutional Ethics Committee. Tonsil specimens were placed on ice immediately after surgical removal. Mononuclear cells (MNC) were isolated by disaggregating tissues in RPMI 1640 medium (Gibco) followed by Ficoll-Isopaque (GE Healthcare) density centrifugation (27). MNC were stained using the following antibodies: anti-CD38-PE (clone HB7, BD Biosciences), anti-IgD-FITC (clone IA6-2, BD Biosciences), anti-CD3-FITC (clone UCHT1, Beckman Coulter), anti-CD184 (CXCR4)-Brilliant Violet 421 (clone 12G5, BioLegend), anti-CD83-APC (clone HB15e, BioLegend). Total GC (CD38^+^/IgD^-^/CD3^-^), DZ (CD38^+^/IgD^-^/CD3^-^/CXCR4^high^/CD83^low^) and LZ (CD38^+^/IgD^-^/CD3^-^/CXCR4^low^/CD83^high^) B cells were sorted using an Influx cell sorter (BD Biosciences). Data rendering was performed using FlowJo (TreeStar).

### Single-Cell Gene Expression and V(D)J Profiling

Cell suspensions were diluted at the concentration of 1,000 cells/μl and analyzed using the Chromium Next GEM Single Cell 5’ kit v1.1, the Chromium Next GEM Single Cell V(D)J Enrichment Kit Human B cell, and the Chromium Controller (10x Genomics), following the manufacturer’s instructions. Sequencing was performed on the NovaSeq6000 System (Illumina). The FASTQ files were aligned to the human GRCh38 reference genome using the 10x Genomics Cell Ranger software v3.1.0 to create unique molecular identifier (UMI) count tables of gene expression for each sample using a pipeline that we developed previously (7). UMI counts were normalized by library size. To annotate cell contig identifier with V(D)J information, the FASTQ files were aligned to the human GRCh38 reference genome using the 10x Genomics Cell Ranger software v3.1.0. The international ImMunoGeneTics information system (IMGT) (28) and the IMGT/HighV-QUEST function were used to assess the V-region identity percentage. The reads were run with IMGT/HighV-QUEST program version 3.5.20 and IMGT/V-QUEST reference directory release 202031-2 (http://www.imgt.org).

A secondary filter step kept only those cells that had both expression and V(D)J information for exactly one heavy and one light chain and were classified by Cell Ranger as productive, full length, with high confidence annotation, and a UMI count greater than 3. We further removed cells with a C-gene classification of IGHD. The mRNA libraries from each specimen displayed a median of 6,052 reads/cell and of 2,112 genes/cell.

Cell Ranger software v3.1.0 (10x Genomics) was also used to identify clonotypes that were defined as: (i) same V(D)J regions and (ii) identical concatenated heavy+light CDR3 nucleotide sequences.

Counts from different patients were merged by log normalizing and scaling the data using the “NormalizeData” and “ScaleData” functions in the Seurat R package (29) along with the “CellCycleScoring” function to regress out the cell cycle effect using the default G2M and S gene sets. The “vars.to.regress” feature of the ScaleData function was used to remove the batch and cell cycle effects. The merged data was dimensionally reduced using Principal Component Analysis (PCA) to get the top 50 principle components using the Scikit-learn Python package (30) PCA function (parameters: n_components=50, and random_state=PCA_RANDOM_STATE).

UMAP 2D projections were created from the PCA data using the UMAP Python package (parameters: n_neighbors=10, mist_dist=0.1, spread=1, and metric=‘correlation’) (31).

The single-cell expression profiles were clustered with the PhenoGraph Python package (32) on the PCA data using the default parameters to build a k-nearest neighbor graph with 20 nearest neighbors (k=20).

The MAST R package was used to test for differentially expressed genes between individual clusters or pairs of clusters (33).

### Pseudo-Temporal Trajectory Analysis

Pseudo-time analysis was performed using the Slingshot R package (34) on the dimensionally reduced data with cells labeled by their PhenoGraph cluster. The cluster trajectories were plotted on top of the merged UMAP projection with nodes placed in the centroid of each cluster.

### Gene Set and Pathway Enrichment Analysis

Pathway enrichment analysis was performed using a hypergeometric test assessing P(X>=N) with a Benjamini-Hochberg false discovery rate correction on the KEGG (c2.cp.kegg.v6.2), BioCarta (c2.cp.biocarta.v6.2), and Hallmark (h.all.v7.0) collections from the Molecular Signature Database (MSigDB) v6.2 (http://software.broadinstitute.org/gsea/msigdb/index.jsp) (35) as well as the Staudt Lab Signature Database (36).

### Statistical Analysis

The MAST R package was used to identify differentially expressed genes in each cluster identified in the sc-RNAseq data. For the gene set and pathway enrichment analysis, a hypergeometric test with a Benjamini-Hochberg false discovery rate correction was used.

Detailed information of the statistical test, number of replicates/samples (defined as n) used in each experiment, and measurement precision are reported in the figure legends. Significance was associated to a p ≤ 0.05.

### Data Availability

Single-cell gene expression data are available from the Gene Expression Omnibus (GEO) database under accession number GSE188617 (https://www.ncbi.nlm.nih.gov/geo/query/acc.cgi?acc=GSE188617).

### Code Availability

All analyses and visualizations were performed in R and Python using the following open-source tools as described above: 10x Genomics Cell Ranger software v3.1.0 (https://support.10xgenomics.com/single-cell-gene-expression/software/pipelines/latest/installation), Seurat (https://satijalab.org/seurat/), PhenoGraph (https://github.com/jacoblevine/PhenoGraph), UMAP (https://github.com/lmcinnes/umap), MAST (https://bioconductor.org/packages/release/bioc/html/MAST.html), DESeq2 (https://bioconductor.org/packages/release/bioc/html/DESeq2.html), Slingshot (https://bioconductor.org/packages/release/bioc/html/slingshot.html/), the Scikit-learn implementations of PCA, the Matplotlib contour function, featureCounts (https://bioconductor.org/packages/release/bioc/html/Rsubread.html), Lifelines (https://github.com/CamDavidsonPilon/lifelines/), seaborn (https://seaborn.pydata.org/index.html), and GSEA (https://www.gsea-msigdb.org/gsea/index.jsp). Figures were created with the svgwrite package (https://pypi.org/project/svgwrite/).

## Results

### Identification of GC B Cell Subpopulations by Single-Cell Transcriptomic Analysis

Human GC B cells were isolated from tonsil tissue of three donors by cell sorting CD3^-^/IgD^-^/CD38^+^ cells. In addition, DZ (CD3^-^/IgD^-^/CD38^+^/CXCR4^high^/CD83^low^) and LZ (CD3^-^/IgD^-^/CD38^+^/CD83^high^/CXCR4^low^) subpopulations were purified in 2 of the 3 donors (**Figure S1A**). Single-cell transcriptomic analysis was performed in parallel with immunoglobulin sequencing using the 10x Genomics Single Cell Immune Profiling Platform (**Figure S1B**). Upon data quality filtering, we obtained 40,772 cells that had good quality data for both the transcriptome and the immunoglobulin sequences. The transcriptome libraries were analyzed by the 10x Genomics computational pipeline, followed by dimensional reduction using the Uniform Manifold Approximation and Projection (UMAP) algorithm (31), and cluster identification by the PhenoGraph algorithm (32) (**Figure S1B**).

**Figure 1:**
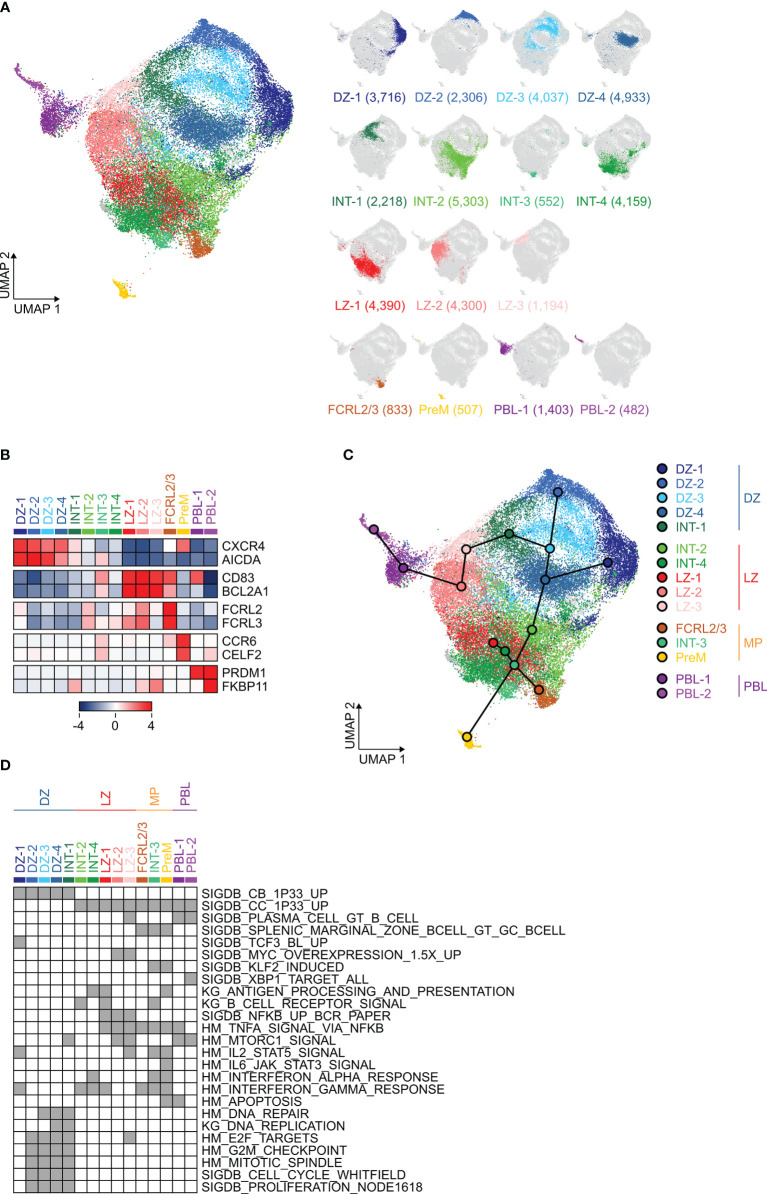
Identification and characterization of germinal center (GC) B cell subpopulations by single-cell (sc)-transcriptomic analysis. **(A)** UMAP projection of sc-RNAseq profiles of 40,772 cells including GC (CD3^−^, IgD^−^, CD38^+^), dark zone (DZ, CD3^−^, IgD^−^, CD38^+^, CD83^lo^, CXCR4^hi^) and light zone (LZ, CD3^−^, IgD^−^, CD38^+^, CD83^hi^, CXCR4^lo^) cells isolated from three donors. Clusters in the UMAP plot were identified by PhenoGraph and color-coded according to different cell states: DZ, Intermediate (INT), LZ, and committed to post-GC differentiation (FCRL2/3; memory precursors, PreM; plasma blasts, PBL). The right panel displays the UMAP projection labeled to display the cells belonging to each cluster. The number of cells in each cluster is provided in parenthesis. **(B)** Heat map displaying the relative expression, as z-scored fold change (log2), of selected hallmark genes in the GC B cell clusters identified in **(A)**. **(C)** Pseudo-time analysis inferred trajectories that were projected onto the UMAP with cluster nodes placed in the centroid of each cluster. **(D)** Pathway enrichment analysis for the gene signatures associated with the clusters identified in **(A)**. Selected pathways from KEGG (KG), Hallmark (HM) and Staudt Lab Signature Database (SigDB) that were significantly enriched (hypergeometric test with Benjamini-Hochberg correction, q < 0.05) are shown in gray. Clusters are organized in four groups (DZ, LZ, memory precursors MP, PBL) based on their similarities, as identified in **(C)**.

Confirming our previous observations (7), 99% of the cells were assigned to clusters that displayed features of DZ, LZ or Intermediate GC B cells as well as clusters representing the precursors of plasma cells and memory B cells (**Figure 1A**). The cluster identities were confirmed by annotation of the cluster-specific gene signatures, which included hallmarks of these subpopulations such as *CXCR4* and *AICDA* (DZ), *CD83* and *BCL2A1* (LZ), *CCR6* and *CELF2* (PreM), *PRDM1* and *FKBP11* (PBL) (**Figure 1B** and **Table S1**). Consistent with a recent report (10), we also identified a cluster of cells (named “FCRL2/3”) displaying some similarities with the memory B cell precursors and characterized by high expression of *FCRL2* and *FCRL3* (**Figures 1A, B** and **Table S1**). As expected, the DZ-sorted cells were mostly associated with the sc-identified DZ clusters, LZ-sorted cells with the LZ compartment and with cells committed to post-GC differentiation, while the GC-sorted cells contributed to all sc-clusters but with a significant preference toward DZ and INT clusters (**Figure S1C**).

In order to infer the relationships across clusters, we applied a pseudo-time analysis that confirmed the proximity of the distinct DZ or LZ clusters, pointed to the relationship between the INT-1, including DZ re-entry cells, and the DZ clusters, and placed INT-3 cells at the switch point toward memory precursors and FCRL2/3 cells (**Figure 1C**). Based on the pseudo-time order and the transcriptional signatures, we identified four major groups of clusters that were labeled as “DZ” (all DZ and the INT-1 clusters), “LZ” (the LZ-like INT-2 and INT-4 clusters and all the LZ clusters), “MP” (the INT-3, PreM and FCRL2/3 clusters) and “PBL” (the PBL-1 and PBL-2 clusters). Of note, although we define cells that display transcriptional similarities with memory B cells as “memory precursors”, we cannot completely exclude that some of them are indeed differentiated memory B cells re-entering the GC reaction or early activated GC B cells which, by retaining some features of naïve B cells, are misclassified as memory-like. In addition, the “PBL” clusters identify GC B cells that acquired transcriptional profiles consistent with commitment to the plasma cell lineage, but we cannot estimate whether these cells will become short-lived plasmablasts or plasma cells.

Pathway enrichment analysis was performed on the transcriptional signatures of each cluster using multiple signature databases (KEGG, Hallmark and the Staudt Lab Signature Database, SigDB). The results confirmed similarities to the centroblast signature for the DZ group and to the centrocyte signature for the other clusters, while more refined signatures highlighted the clusters associated with post-GC differentiation (**Figure 1D** and **Table S2**). Several transcriptional signatures associated with transcription factors active at specific stages of the GC reaction were consistently identified in the expected clusters: TCF3 in DZ cells (DZ-1), MYC in activated LZ cells (LZ-2 and LZ-3), KLF2 in cells committed to memory B cell differentiation (INT-3 and PreM), XBP1 in plasma blasts (PBL-2) (**Figure 1D** and **Table S2**).

Overall, this collection represents the largest dataset of human GC B cell sc-transcriptomic data to date and provides a detailed characterization of GC B cell subpopulations, in line with previous studies by us (7) and others (8–13).

### Tracking the Immunoglobulin Variable Region Mutation Load in GC B Cell Subpopulations

**Figure 2:**
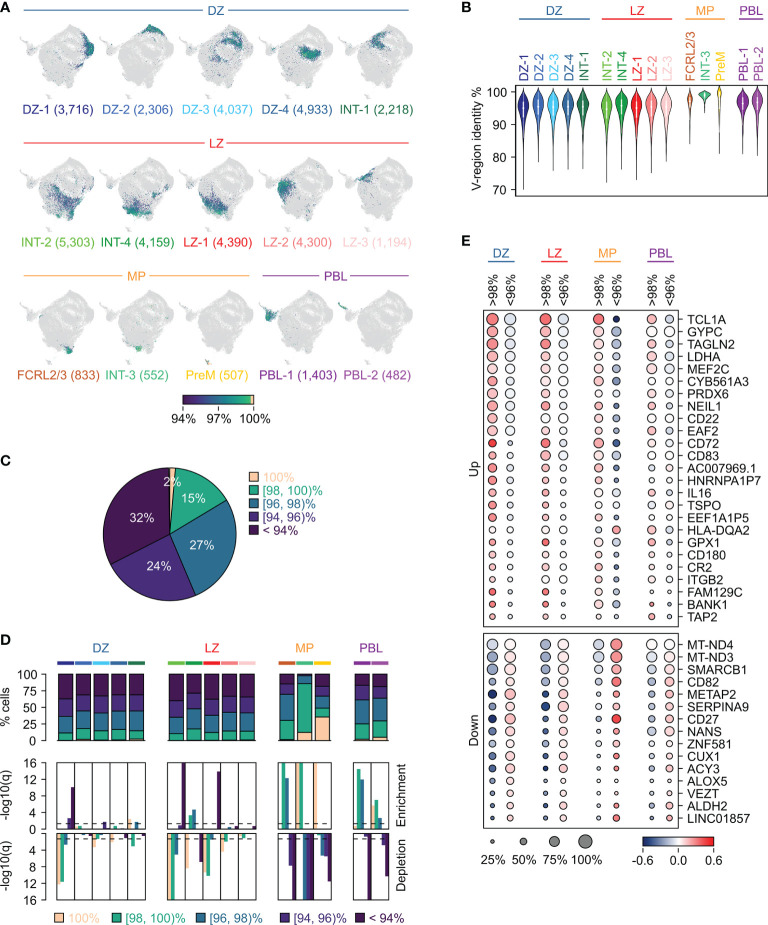
Tracking the immunoglobulin variable region mutation load in GC B cell subpopulations. **(A)** UMAP projection in which cells in each cluster are labeled based on the germline identity of the variable (V)-regions of the heavy and light immunoglobulin chains. The color scale reflects the percentage of the V-region germline identity with a range from 100% (peach) to 94% or less (dark blue). The number of cells belonging to each cluster is provided in parenthesis. Clusters are organized in four groups (DZ, LZ, memory precursors MP, PBL) based on their similarities. **(B)** Violin plots showing the distribution of the V-region germline identity in each GC cluster. **(C)** Distribution of GC B cells into five subgroups based on the V-region germline identity. **(D)** Stacked bar plot showing for each GC cluster the percentage of cells within the V-region germline identity subgroups (top panel). Enrichment (middle panel) and depletion (bottom panel) analyses of the mutational load in the GC clusters (hypergeometric test with Benjamini-Hochberg correction, q < 0.05). **(E)** Differential gene expression analysis between cells with high (>98%) and low (<96%) V-region germline identity in each of the major GC groups (DZ, LZ, MP and PBL). The size of the dot indicates the percentage of cells with detectable expression, and the color reflects the relative gene expression shown as the z-scored average (log2) normalized expression within a group.

Analysis of the mutations physiologically introduced by the SHM mechanism in the variable (V) regions of the heavy and light immunoglobulin chains was performed at single-cell level using the 10x Genomics Single Cell Immune Profiling Platform. As expected, the majority of GC B cells carried mutations in their immunoglobulin V regions (germline identity median: 95.5%; range: 71-100%) (**Figures 2A, B** and **Table S3**). Only 2% of cells expressed completely unmutated BCRs (germline identity=100%; **Figure 2C**). The overall mutational load was variable with a minority of cells (15%) displaying high germline identity (>98%), while 32% of cells carried very heavily mutated V regions (germline identity <94%) (**Figure 2C**). The clusters associated with the DZ and LZ compartments, as defined by the analysis of the sc-transcriptome (**Figure 1**), displayed an overall similar range of mutations (**Figures 2A, B**), with a few clusters (DZ-1, INT-2 and LZ-1) biased toward a higher mutational load by displaying a significant enrichment for cells with a germline identity lower than 94% (**Figure 2D**).

The clusters associated with the memory precursors (“MP” group: FCRL2/3, INT-3 and PreM) were enriched in cells displaying a very low mutational load (germline identity >98%), with the INT-3 population being almost exclusively represented by cells with a germline identity >98% (**Figure 2D**). Although the PreM cluster was significantly enriched for unmutated cells, which accounted for 36% of the cells in this cluster, 51% of the PreM cells were significantly mutated (germline identity <98%; range: 83-98%; median: 95.1%) (**Figures 2A, B, D**).

About 75% of the cells in the PBL clusters carried a mutated BCR (germline identity <98%; range: 82.3-98%; median: 95.8%). Nonetheless, the PBL clusters were overall depleted for heavily mutated cells (germline identity <94%), which represented less than 17% of the population compared to the average 34% detected in the DZ and LZ groups (**Figure 2D**).

In order to identify genes the expression of which correlates with the V-region mutational load, we compared cells with low (germline identity >98%) and high (germline identity <96%) number of mutations in each of the cluster subgroups. A subset of differentially expressed genes displayed the same trend in multiple groups, regardless of their GC state and isotype class (**Figures 2E**, **S2**). Among them, genes involved in the BCR signaling pathway (*CD72*, *CR2*, *CD22*, *BANK1*, *TCL1A*) and the BCR-responsive *MEF2C* transcription factor displayed higher expression in cells with a low mutational load (germline identity >98%). Consistent with the fact that cells with high V-region germline identity may display lower affinity for the antigen, several of these markers were previously shown to be expressed at higher level in low-affinity mouse GC B cells compared to high-affinity cells (20). Conversely, and as previously reported (10), the B cell maturation marker *CD27* was expressed at higher levels in cells with a high mutational load (germline identity <96%). In addition, cells with a high mutational load (germline identity <96%) displayed higher expression of *CUX1*, a repressor of immunoglobulin transcription (37), of the SWI/SNF member *SMARCB1*, and of genes encoding the mitochondrial Complex I (**Figure 2E**).

In order to identify the presence of clones originating from the same B cell precursor, clonotype analysis was performed on each donor separately. Although the large majority of cells (62.3%-85.8%) displayed unique clonotypes, clones with the same clonotype were identified in all patients (**Figure S3**). The number as well as the size of the clones were variable across patients but overall proportional to the number of cells analyzed (**Figure S3**). Clonal populations displayed no significant enrichment relative to the GC B cell subpopulations: they were uniformly distributed, proportionally to the size of each cluster.

In conclusion, our analysis provides a detailed overview of the V-region mutational pattern in GC B cells that highlights a remarkably homogeneous distribution of mutational loads across different subpopulations, supports the presence of two PreM populations characterized by low and high germline identity, respectively, and demonstrates the lower V-region mutational load of cells committed to plasma cell differentiation relative to the bulk of GC B cells.

### Immunoglobulin Isotype Class Distribution in GC B Cell Subpopulations

**Figure 3:**
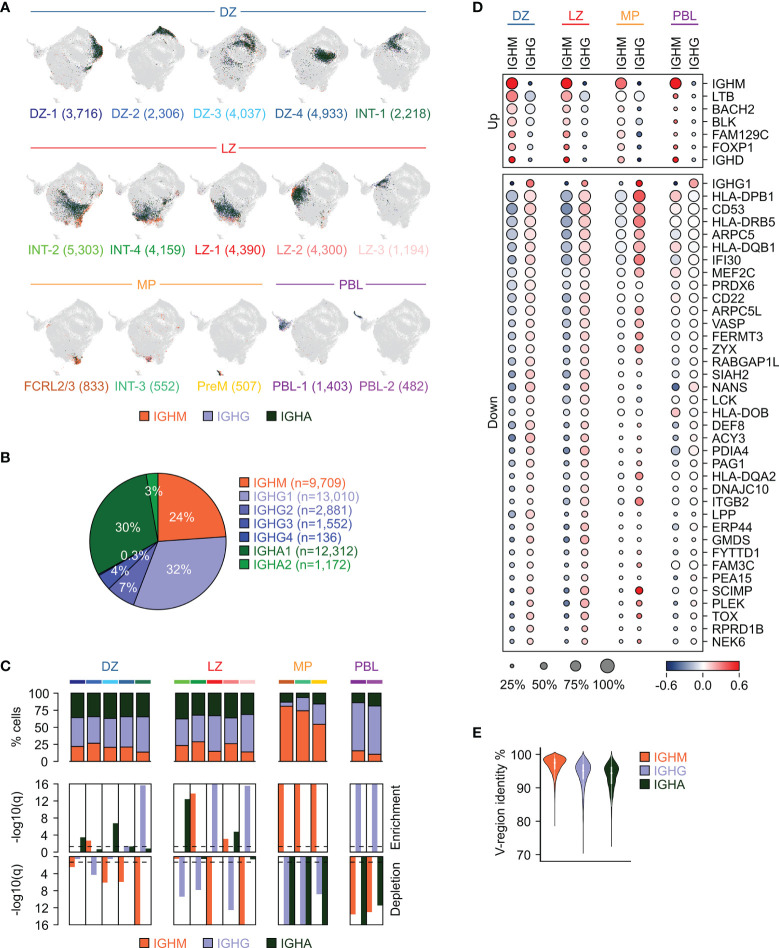
Immunoglobulin isotype distribution in GC B cell subpopulations. **(A)** UMAP projection in which cells in each cluster are labeled based on the immunoglobulin isotype class type: IGHM (orange), IGHG (light blue), or IGHA (dark green). The number of cells belonging to each cluster is provided in parenthesis. **(B)** Distribution of GC B cells based on their expression of isotype subclasses. **(C)** Stacked bar plots showing for each GC cluster the percentage of cells within the immunoglobulin isotype classes (top panel). Enrichment (middle panel) and depletion (bottom panel) analyses of the immunoglobulin isotype classes in the GC clusters (hypergeometric test with Benjamini-Hochberg correction, q < 0.05). **(D)** Differential gene expression analysis between unswitched (IGHM) and switched (IGHG) cells in each of the major GC subgroups (DZ, LZ, MP and PBL). The size of the dot indicates the percentage of cells with detectable expression, and the color reflects the relative gene expression shown as the z-scored average (log2) normalized expression within a group. **(E)** Violin plot showing the distribution of the V-region identity in cells expressing unswitched (IGHM) or switched (IGHG and IGHA) immunoglobulin isotype classes.

The GC data obtained with the 10x Genomics Single Cell Immune Profiling Platform also allowed the identification of the IGH constant region expressed in each cell. Except for IGHE, all IGH isotype classes were detected in each of the GC B cell clusters (**Figure 3A** and **Table S3**). Among them, the IGHG class (including IGHG1, IGHG2, IGHG3, and IGHG4) was the most common, accounting for about 43% of the cells, followed by IGHA (33%, including IGHA1 and IGHA2), with IGHM cells representing the remaining 24% of the total GC B cells (**Figure 3B**).

The DZ, LZ and the related intermediate cells (DZ and LZ groups) displayed an overall similar distribution with IGHG and IGHA representing on average 42% (range 39-45%) and 35% (range 34-37%) of the cells, respectively (**Figure 3C**). A few clusters showed a bias in favor of either class with a significant enrichment for IGHG observed in the DZ re-entry cells (INT-1) and in a subset of the LZ cells (LZ-1 and LZ-3), while IGHA-expressing cells were enriched in DZ-1, DZ-3, INT-2 and LZ-2 (**Figure 3C**). IGHM-positive cells represented 21% (range 14-29%) of the DZ, LZ and the associated intermediate compartments, while they became dominant (median: 74%; range 54-81%) in cells related to the memory precursors (FCRL2/3, INT-3 and PreM clusters) and depleted in the PBL, which were instead enriched for IGHG cells (median: 71%) (**Figure 3C**).

Differential expression analyses comparing switched and unswitched cells in each of the major subgroups (DZ, LZ, MP and PBL) identified genes displaying consistent, significant difference in at least two of the groups (**Figure 3D**). A few genes displayed higher expression in the unswitched cells, including the BCR-associated signaling molecule *BLK*, lymphotoxin beta *LTB*, and the transcription factors *BACH2* and *FOXP1*. Conversely, a much larger set of genes was induced in the switched cells including genes involved in antigen presentation (*SCIMP*, *IFI30*, and several HLA molecules), BCR signaling (*CD22*, *CD53*, *MEF2C*) and focal adhesion (*ITGB2*, *FERMT3*, *VASP*, *ARPC5*, *ARPC5L1*) (**Figure 3D**). In addition, we identified *TOX*, a high mobility group box protein that has been reported to be upregulated in switched memory B cells (38), to be induced in the large majority of switched cells in the GC including DZ, LZ and PreM (**Figure 3D**). Of note, the genes displayed in **Figure 3D** are associated with the specified isotype class regardless of the mutational load in the variable regions. However, since low IGV mutational load tends to correlate with expression of unswitched IGH (**Figures 3E**, **S4**), a large fraction of genes that are differentially expressed between switched and unswitched cells are also associated with the IGHV mutational load. In order to dissect these two aspects (mutational load vs isotype) the differential expression analyses were performed separately, according to the IGHV mutational load, and the results are reported in **Figure S5**. Consistent with previous observations (39), *CXCR5*, a chemokine receptor associated with LZ migration (2), was expressed at higher levels in IGHM-positive cells that displayed high germline identity (>98%). Among the genes expressed at higher level in IGHM cells with high IGV mutational load (germline identity <96%) we detected *FCRL2*, which although representing a hallmark of a specific GC subpopulation (FCRL2/3), also appeared to be expressed in a small fraction of other populations (**Figure S5**).

Overall, these results indicate that the large majority of GC B cells show a similar pattern of isotype distribution dominated by switched cells; however, cells committing to memory B cell or plasma cell differentiation display unique repertoires characterized by enrichment for IGHM and IGHG, respectively.

### GC Precursors of Memory B Cells and Plasma Cells Display Immunoglobulin Repertoires Distinct From the Other GC B Cell Populations

**Figure 4:**
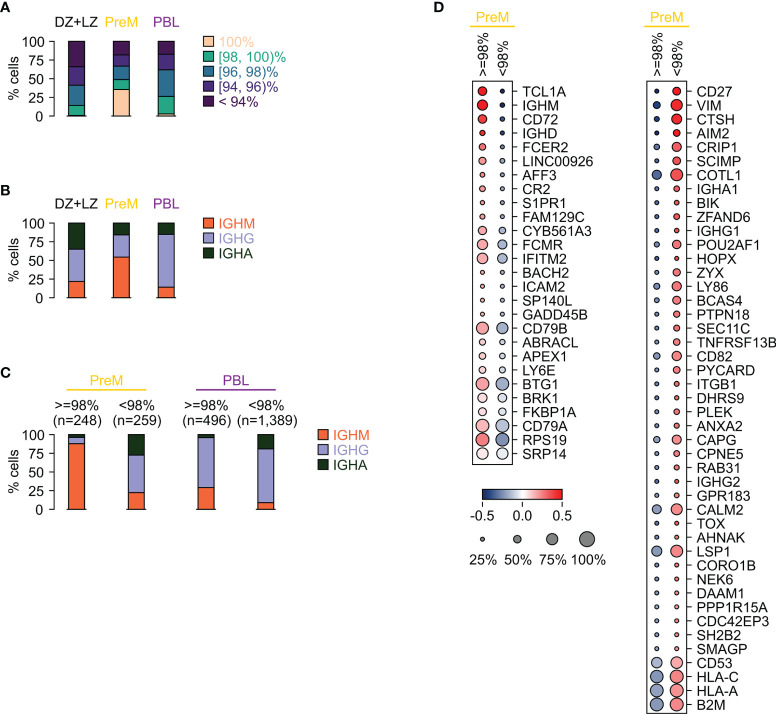
PBL and PreM display unique immunoglobulin repertoires. **(A)** Stacked bar plot showing for the indicated cell populations, GC (DZ+LZ groups), PreM and PBL (PBL1+PBL2 clusters), the percentage of cells within the V-region germline identity subgroups. **(B)** Stacked bar plots showing for the indicated cell populations the percentage of cells within the immunoglobulin isotype classes. **(C)** Stacked bar plots showing for the PreM and PBL populations the percentage of cells within the immunoglobulin isotype classes in the fractions with high (≥98%) and low (<98%) V-region germline identity. **(D)** Heat map displaying the differentially expressed genes between PreM cells with high (≥98%) and low (<98%) V-region germline identity. The size of the dot indicates the percentage of cells with detectable expression, and the color reflects the relative gene expression shown as the z-scored average (log2) normalized expression. Only genes displaying a fold change ≥1.2 and detectable expression in at least 25% of the cells in either group are depicted.

As described above, PreM and PBL showed an overall lower fraction of cells carrying highly mutated V-regions with only 18% of PreM and 17% of PBL displaying a V-region germline identity < 94% compared to 34% of the bulk GC B cells (**Figure 4A**). Whereas in PreM the skewing could be due to the increased fraction of cells displaying high germline identity, this does not apply to PBL.

PBL and PreM express distinct IGH isotype classes: IGHM is predominantly associated with PreM (54%) and IGHG with PBL (71%) (**Figure 4B**). PBL and plasma cells have already been shown to predominantly express an IGHG isotype and it has been suggested that the signaling pathways related to IGHG may contribute to plasma cell differentiation (10, 25, 40, 41).

Although significantly enriched for IGHM-expressing cells, 46% of PreM cells expressed IGHG (30%) or IGHA (16%), consistent with the presence of at least two types of memory precursors: unswitched and switched (**Figure 4B**). While PBL mostly expressed IGHG isotypes in cells with high or low mutational load, PreM displayed a strong correlation between V-region germline identity and IGH isotypes with low mutational load paired with IGHM expression and high mutational load associated with a switched isotype (**Figure 4C**).

These two subgroups of PreM displayed similar expression of GC PreM markers such as *CCR6*, *BANK1*, *RASGRP2* and *CELF2*. However, a subset of genes was differentially expressed and included genes that were related to the V region mutational load (i.e. *TCL1A*, *CD72*, *CR2*, *CD27*, *CD82*) or isotype classes (i.e. *SCIMP*, *ZYX*, *TOX*, *BACH2*) (**Figure 4D**). Of note, these signatures were detected in multiple GC subpopulations, in addition to PreM, suggesting that some of the differences were driven only by the antibody repertoire (**Figure 2E**, **3D**, **4D**). Nonetheless, intrinsic differences were also detected between these two PreM subpopulations, with the group associated with high germline identity and unswitched IGH showing overall higher expression of BCR components (*CD79A* and *CD79B*), of the quiescence-associated gene *BTG1*, and of the *S1PR1* receptor that favors migration toward the marginal zone (**Figure 4D**). Switched and highly mutated PreM displayed higher expression of *TNFRSF13B*, of the activation marker *GPR183*, of the tetraspan molecules *CD82* and *CD53*, and of MHC class I molecules (*HLA-A*, *HLA-B*, *HLA-C*, *B2M*).

Overall, these results are consistent with current evidence supporting the development of distinct memory B cell populations with low and high mutational load, which are primed for early GC re-entry or for long lasting memory, respectively (42, 43).

### Expression of Genes Implicated in Somatic Hypermutation and Class Switch Recombination Is Induced in Specific GC B Cell Populations

**Figure 5:**
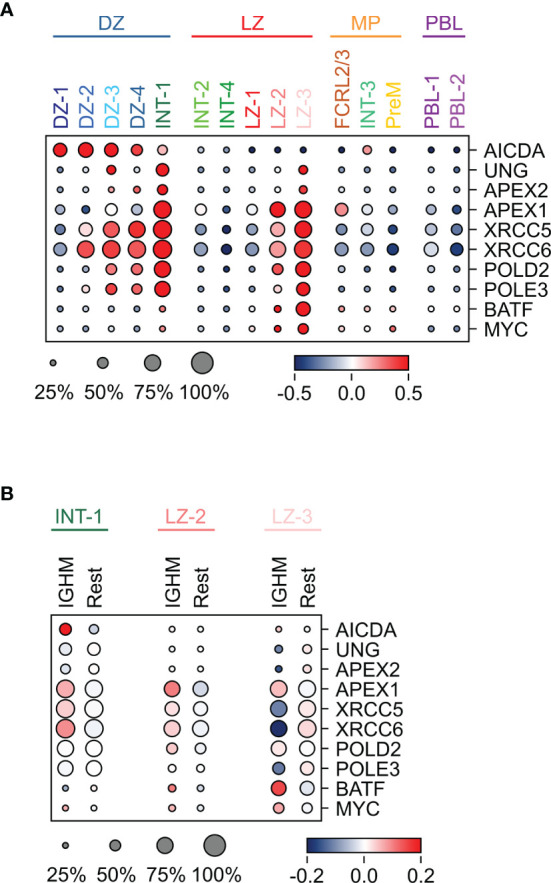
Expression pattern of genes implicated in SHM and/or CSR in GC B cell populations. **(A)** Heat map displaying the expression of genes implicated in SHM and CSR across the GC populations, identified by sc-transcriptomics. **(B)** Heat map displaying the expression of the same genes analyzed in **(A)**, in unswitched (IGHM) and switched (Rest) cells in the INT-1, LZ-2 and LZ-3 GC clusters. The size of the dot indicates the percentage of cells with detectable expression, and the color reflects the relative gene expression shown as the z-scored average (log2) normalized expression within a cluster.

The mechanism of SHM introduces mutations in the immunoglobulin variable regions to enhance the diversity of the encoded receptor. This process is known to occur in the DZ and it starts with a cytosine deamination process catalyzed by AICDA followed by error-prone repair mechanisms (44). Consistently, *AICDA* was induced in the DZ clusters, while the N-glycosylase *UNG* and the endonuclease *APEX2*, known to play a role in SHM (45, 46), were co-expressed at the highest levels in the DZ-3, consisting almost exclusively of cells in S-G2-M, and in the INT-1 population that identifies the cells exiting or re-entering the DZ, suggesting that the repair process may extend beyond the DZ compartment (**Figure 5A**). The expression pattern of these key players support the current view of SHM occurring in the DZ compartment.

Although the process of CSR has been dissected in detail at the molecular level (23), in which cells it occurs remains elusive. Several lines of evidence in mouse and human cells suggested that CSR occurs in B cells before entering the GC reaction (10, 24–26). Here, we detected high expression of genes involved in CSR, including *APEX1* (47), *UNG* (48–50), *XRCC5/6* (51, 52), *POLD2*, *POLE3*, and *BATF* (53), in a few GC subpopulations, suggesting that a subset of cells may undergo CSR in the GC. In particular, *BATF* that is required for the expression of germline transcripts and for effective CSR (53), was highly induced in a fraction of LZ-2 and in the majority of LZ-3 cells (**Figure 5A**). In addition, *APEX1* was upregulated in the majority of LZ-2 and LZ-3 populations, which represent the most activated LZ cells, and in the INT-1 population, which includes the DZ exit/re-entry cells (**Figure 5A** and **Table S1**). INT-1 cells expressed high levels of *AICDA*, which remained clearly expressed in the LZ-related intermediate populations (INT-2 and INT-4), but was significantly downregulated in the LZ cells (**Figure 5A**). The subset of LZ GC B cells with the highest expression of CSR-related genes, is represented by cells that, based on their transcriptome changes, are undergoing strong T-cell mediated activation and display down-regulation of *BCL6*, known to impair CSR to IGHE (54, 55), and induction of *MYC*, a marker of positive selection and a transcriptional target of BCL6 (56, 57) (**Figure 5A**). These activation signals are known to associate with CSR and may contribute to the opening of specific switch regions.

In the INT-1 and LZ-2 subpopulations, the CSR-related genes displayed a tendency for higher expression in IGHM-positive cells compared to switched cells, suggesting a preferential induction of genes associated with the CSR machinery in IGHM-positive cells (**Figure 5B**). Conversely, the LZ-3 population displayed a homogenous higher expression of most CSR-related genes, regardless of the class isotype.

Consistent with the possibility that CSR may occur during the GC reaction, about 21% of the identified clonotypes (with at least 5 cells, **Figure S3**) included cells harboring different isotypes.

Overall, these data are consistent with the presence of SHM activity in DZ GC B cells, suggest that CSR may occur in a subset of LZ cells, and highlight the possibility that both processes may take place in cells exiting and/or re-entering the DZ (INT-1 population).

## Discussion

Our single cell analysis of the transcriptome and antibody repertoire provides a snapshot of GC B cells in the context of chronic antigenic stimulation. To date, this dataset is the largest collection of sc-transcriptomic data specifically focused on GC B cells, representing a valuable resource for other studies in the field. Consistent with previous observations from us (7) and others (8–13), our transcriptomic analysis identified multiple subpopulations expanding the classic DZ/LZ view into a gradient of states including intermediate subpopulations, which appeared to be related to either the DZ, the LZ or cells committing to post-GC differentiation. In addition, GC precursors of memory B cells and plasma cells were promptly identified by their unique transcriptome.

The paired analysis of the transcriptome and antibody repertoires provided substantial evidence that the large majority of GC B cell subpopulations display similar patterns of V-region mutational load and isotype class distribution. This observation suggests that, at steady-state, GC B cells have the same probability to undergo SHM and that the overall isotype representation remains uniform across distinct DZ, intermediate and LZ subpopulations with a dominant presence of switched cells. Conversely, cells committed to post-GC differentiation displayed unique and distinct behaviors.

Consistent with previous observations on mature memory B cells in the periphery (58–60), GC memory B cell precursors appeared to be split in two subgroups based on both their V-region germline identity and switched or unswitched isotype class. On one side, memory precursors with high germline identity and expressing prevalently IGHM are likely to differentiate in the subset of memory B cells, which will re-enter future GC-reactions (43). On the other, about 50% of memory precursors were switched and displayed an IGV mutational pattern similar to PBL: these cells may generate the memory B cell population that, during secondary immune responses, is able to differentiate into antibody-secreting plasma cells (42).

As expected, plasma cell precursors were mostly mutated and largely expressed IGHG. However, PBL displayed an IGV mutational pattern distinct from the majority of GC B cells and characterized by an overall under-representation for cells with very high mutational load. Considering that plasma cells exiting the GC secrete high-affinity antibodies, these observations suggest that high affinity may not be necessarily achieved by a high load of V-region mutations. Regarding the isotype class, we observed a significant enrichment for IGHG expression in cells committed to plasma cell differentiation, in line with the fact that IGHG-positive GC B cells have been shown to undergo differentiation into plasma cells more efficiently than IGHM-positive cells (39, 41).

Historically, the GC compartment has been considered the site where SHM and CSR occur in the context of T-cell dependent immune responses. Our observations are consistent with the large body of data demonstrating that SHM occurs in DZ GC B cells. GC B cells have also been thought to undergo CSR, a mechanism acting in concert with SHM to give rise to a diverse repertoire of high-affinity antibodies of multiple isotype classes. However, evidence in mouse and human cells suggested that CSR mostly occurs in B cells before entering the GC reaction (10, 24–26). In addition, constant region-based positive selection has been proposed to occur in the GC, independently of CSR (41). Although our data do not address the events occurring in pre-GC B cells, they detected abundant expression of genes associated with the CSR machinery in selected GC subpopulations, including LZ cells and GC B cells exiting and/or re-entering the DZ (INT-1). These data support the presence of the CSR machinery in a small subset of activated GC B cells and suggest that CSR may also occur during the GC reaction.

In conclusion, these data, together with the analysis of the overall B cell compartment in the secondary lymphoid organs (10), provide a framework to integrate observations at single-cell level from mouse models (11–13) and to address still open questions regarding the physiology of the GC reaction, including the fate decision of post-GC effector cells, as well as the pathologies associated with this compartment.

## Data Availability Statement

The datasets presented in this study can be found in online repositories. The names of the repository/repositories and accession number(s) can be found in the article/**Supplementary Material**.

## Author Contributions

RD-F and KB designed and supervised the study. CC, QS, and KB performed single-cell RNA-seq experiments. AH performed computational analyses. CC, AH, LP, and KB analyzed data. EG collected normal lymphoid tissue. CC, AH, LP, RD-F, and KB wrote the paper. All authors read, reviewed and approved the final manuscript.

## Funding

This study was supported by N.I.H. grant R35CA-210105 (to RD-F), by a 10x Genomics/Illumina Pilot Award (to KB) and funded in part through the NIH/NCI Cancer Center Support grant P30CA013696. This research used the resources of the Cancer Center Flow Core Facility (Columbia University), the Genomics and High Throughput Screening Shared Resource (Columbia University) and the Human Immune Monitoring Core (Columbia University).

## Conflict of Interest

The authors declare that the research was conducted in the absence of any commercial or financial relationships that could be construed as a potential conflict of interest.

## Publisher’s Note

All claims expressed in this article are solely those of the authors and do not necessarily represent those of their affiliated organizations, or those of the publisher, the editors and the reviewers. Any product that may be evaluated in this article, or claim that may be made by its manufacturer, is not guaranteed or endorsed by the publisher.
