# Supplementary material for: Tracking Immunoglobulin Repertoire and Transcriptomic Changes in Germinal Center B Cells by Single-Cell Analysis
Source: Front Immunol. 2022 Jan 12;12:818758. doi: 10.3389/fimmu.2021.818758 (PMC8789751; doi:10.3389/fimmu.2021.818758)
Supplement: Supplementary file 1 [file Image_1.pdf]

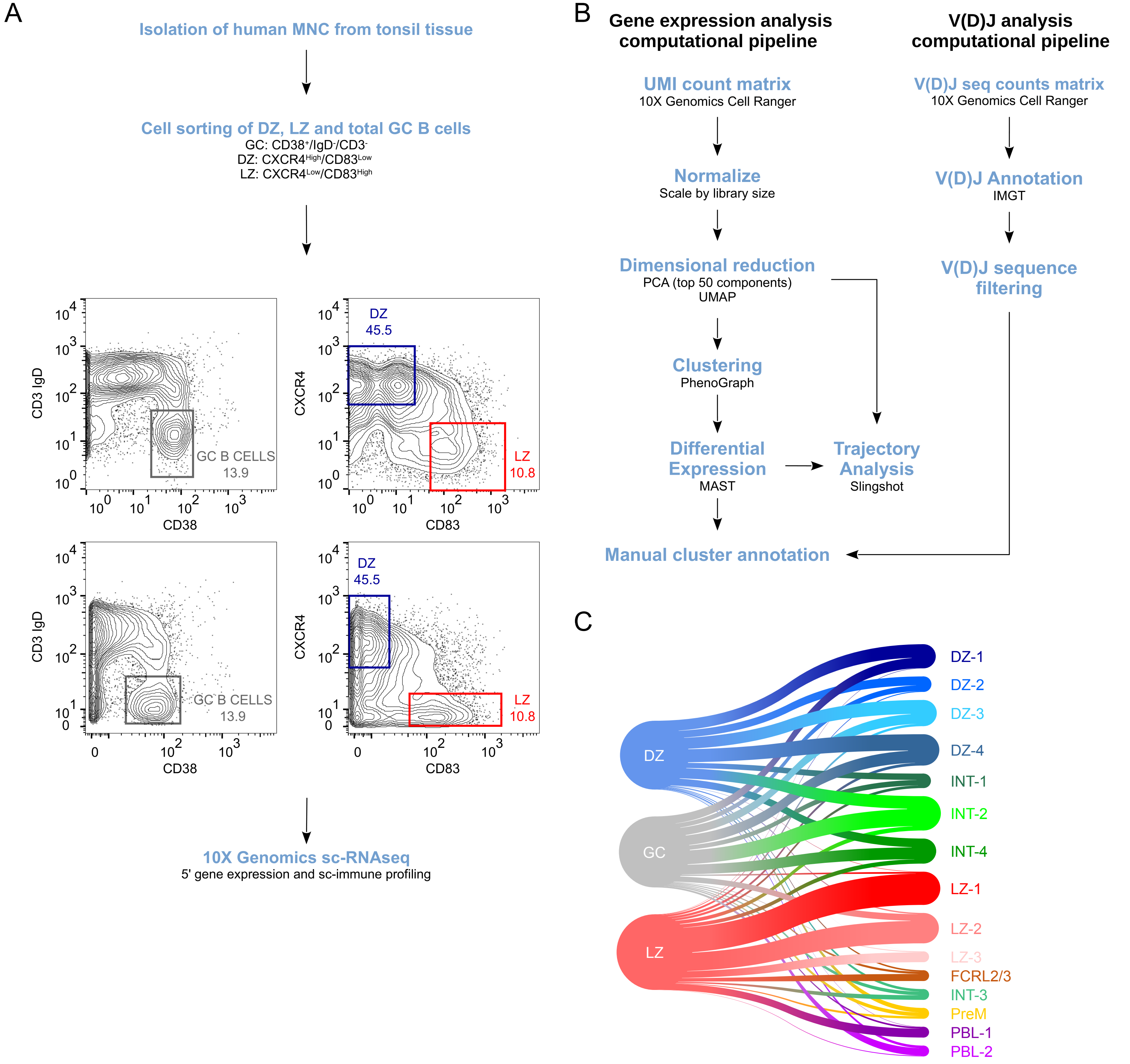

Supplementary Figure 1. Identification of germinal center B cell subpopulations by single-cell transcriptomic: experimental and computational pipelines. (A) Human germinal center (GC) B cells were isolated from mononucleated cells (MNC) from tonsil tissue of three donors by cell sorting CD3$^-$/IgD$^-$/CD38$^+$ cells, while dark zone (DZ) and light zone (LZ) B cells were purified based on the expression of the CXCR4 and CD83 markers GC cells. Representative counterplots and gating strategy from cytofluorimetric analysis of human GC (CD3$^-$/IgD$^-$/CD38$^+$), DZ (CD3$^-$/IgD$^-$/CD38$^+$/CD83^{lo}/CXCR4^{hi}), and LZ (CD3$^-$/IgD$^-$/CD38$^+$/CD83^{hi}/CXCR4^{lo}) B cells isolated from tonsil tissue are shown. The same specimen and analyses are displayed using a log (top panels) or hyperlog (bottom panels) transformation. **(B)** Scheme of the computational steps followed to analyze the single cell (sc)-RNAseq and the V(D)J sequencing data, indicating the software tools used (black labels). **(C)** Summary of the relationship between FACS-sorted DZ, GC and LZ B cells and their cluster assignment by sc-transcriptomic analysis. The analysis includes data from the two patients for which all populations were sorted.
