# Supplementary material for: Tracking Immunoglobulin Repertoire and Transcriptomic Changes in Germinal Center B Cells by Single-Cell Analysis
Source: Front Immunol. 2022 Jan 12;12:818758. doi: 10.3389/fimmu.2021.818758 (PMC8789751; doi:10.3389/fimmu.2021.818758)
Supplement: Supplementary file 3 [file Image_3.pdf]

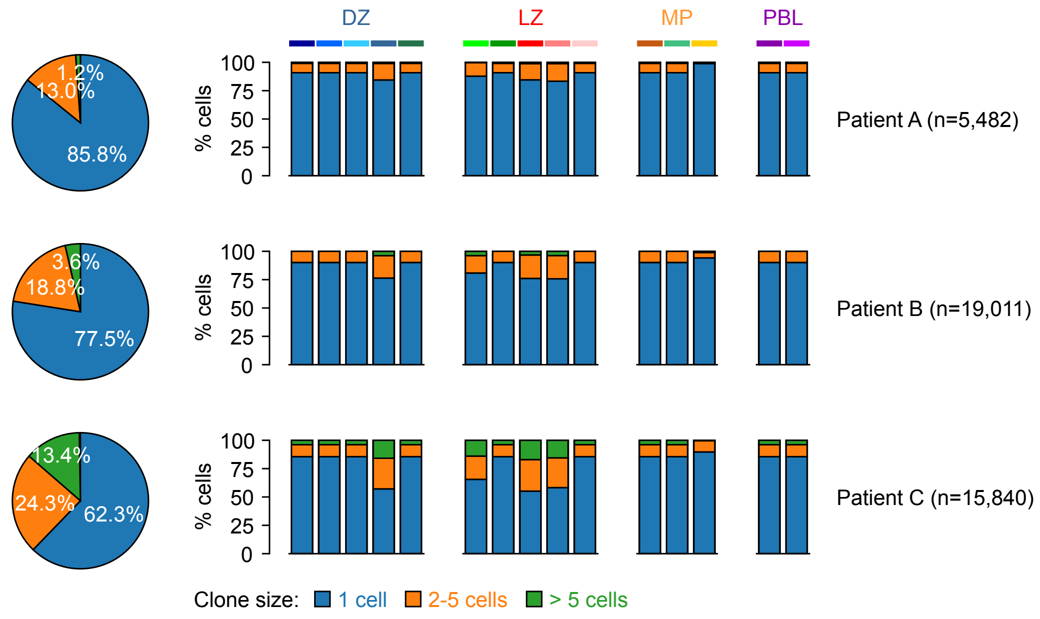

Supplementary Figure 3. Clonotype analysis. Pie charts (left) report the percentage of cells in each patient that display unique or shared clonotypes. Stacked bar plots (right) display the distribution of cells with unique or shared clonotypes in the GC B cell subpopulations. Cells are grouped based on the size of their clonotype clone.
