# Supplementary material for: Tracking Immunoglobulin Repertoire and Transcriptomic Changes in Germinal Center B Cells by Single-Cell Analysis
Source: Front Immunol. 2022 Jan 12;12:818758. doi: 10.3389/fimmu.2021.818758 (PMC8789751; doi:10.3389/fimmu.2021.818758)
Supplement: Supplementary file 4 [file Image_4.pdf]

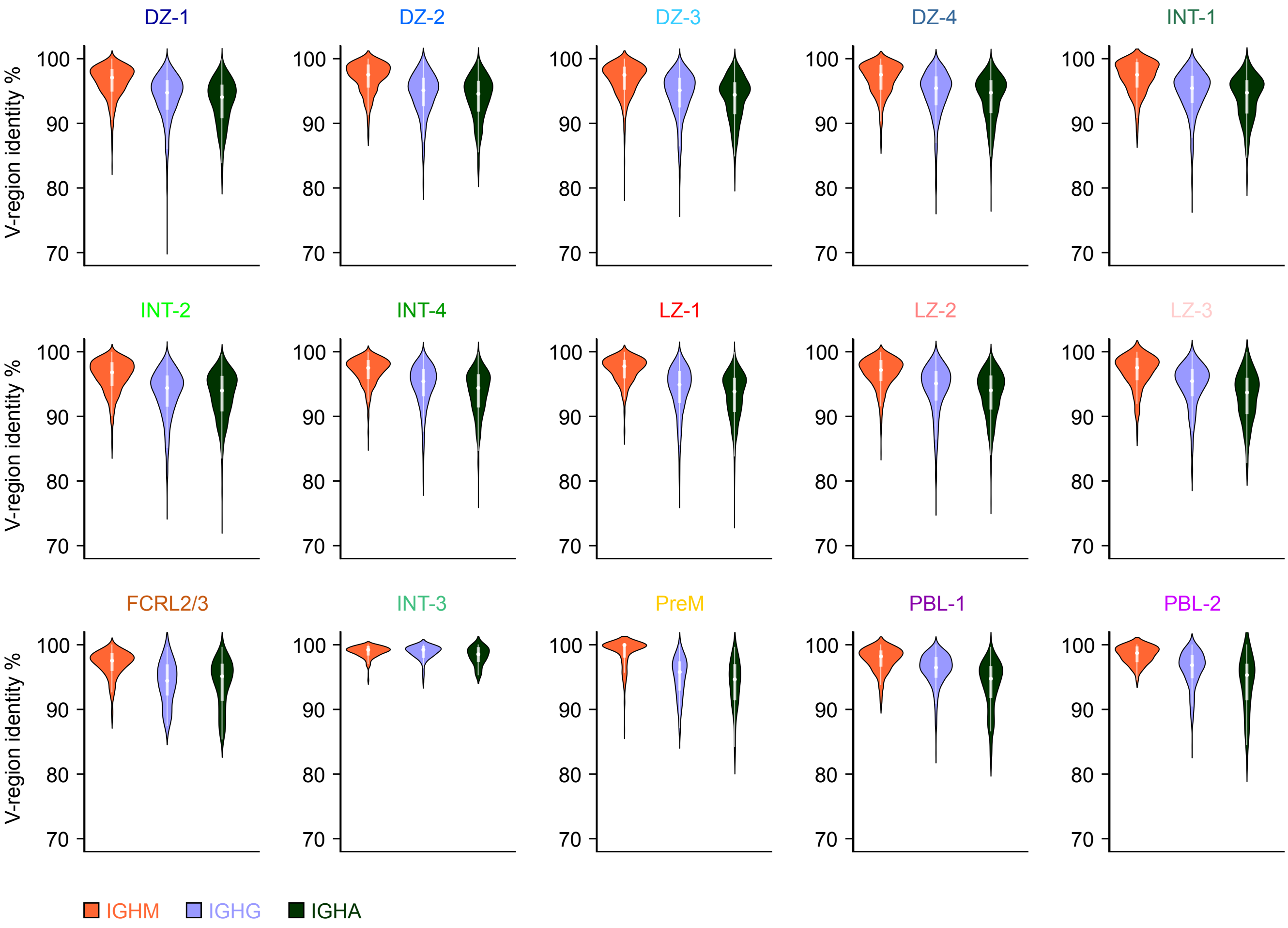

Supplementary Figure 4. Relationship between variable region germline identity and isotype classes. Violin plots showing the V-region identity distribution in cells within each GC subpopulation, grouped by their isotype class.
