# Supplementary material for: Tracking Immunoglobulin Repertoire and Transcriptomic Changes in Germinal Center B Cells by Single-Cell Analysis
Source: Front Immunol. 2022 Jan 12;12:818758. doi: 10.3389/fimmu.2021.818758 (PMC8789751; doi:10.3389/fimmu.2021.818758)
Supplement: Supplementary file 5 [file Image_5.pdf]

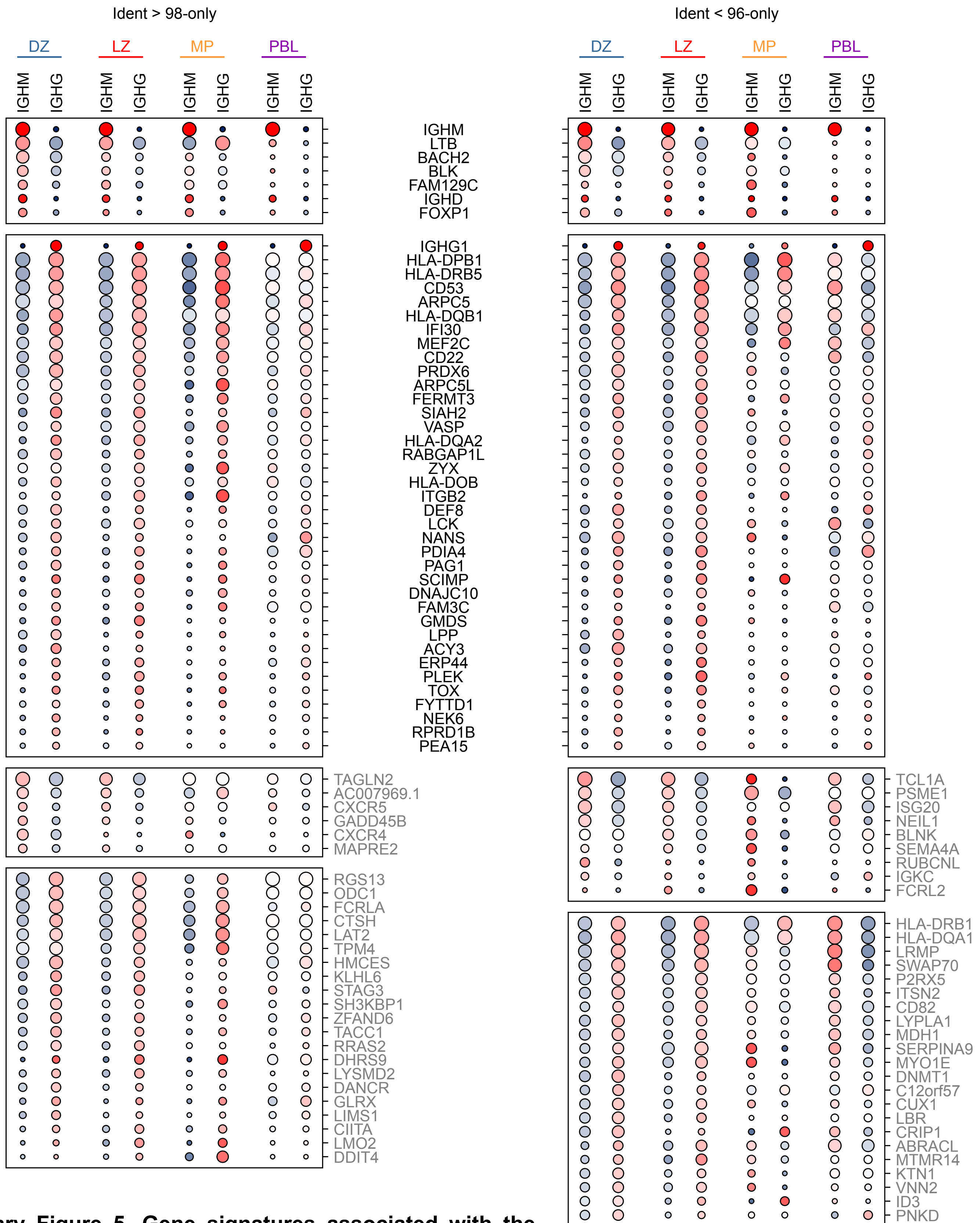

Supplementary Figure 5. Gene signatures associated with the isotype classes in the major GC subgroups. Differential gene expression analysis between GC unswitched (IGHM^{+}) and switched (IGHG^{+}) B cells in each of the major GC subgroups (DZ, LZ, MP and PBL). The analysis was performed separately in cells with high (>98%; left panel) or low (<96%; right panel) V-region germline identity. Genes that were significantly differentially expressed in at least two subgroups are displayed: the upper panel (black labels) shows genes with a consistent pattern in both low and high germline identity groups, while the lower panel (gray labels) shows genes detected only in one of the analyses. The size of the dot indicates the percentage of cells with detectable expression, and the color reflects the relative gene expression shown as the z-scored average log2 normalized expression within a group.
