# Supplementary material for: Tracking Immunoglobulin Repertoire and Transcriptomic Changes in Germinal Center B Cells by Single-Cell Analysis
Source: Front Immunol. 2022 Jan 12;12:818758. doi: 10.3389/fimmu.2021.818758 (PMC8789751; doi:10.3389/fimmu.2021.818758)
Supplement: Supplementary file 2 [file Image_2.pdf]

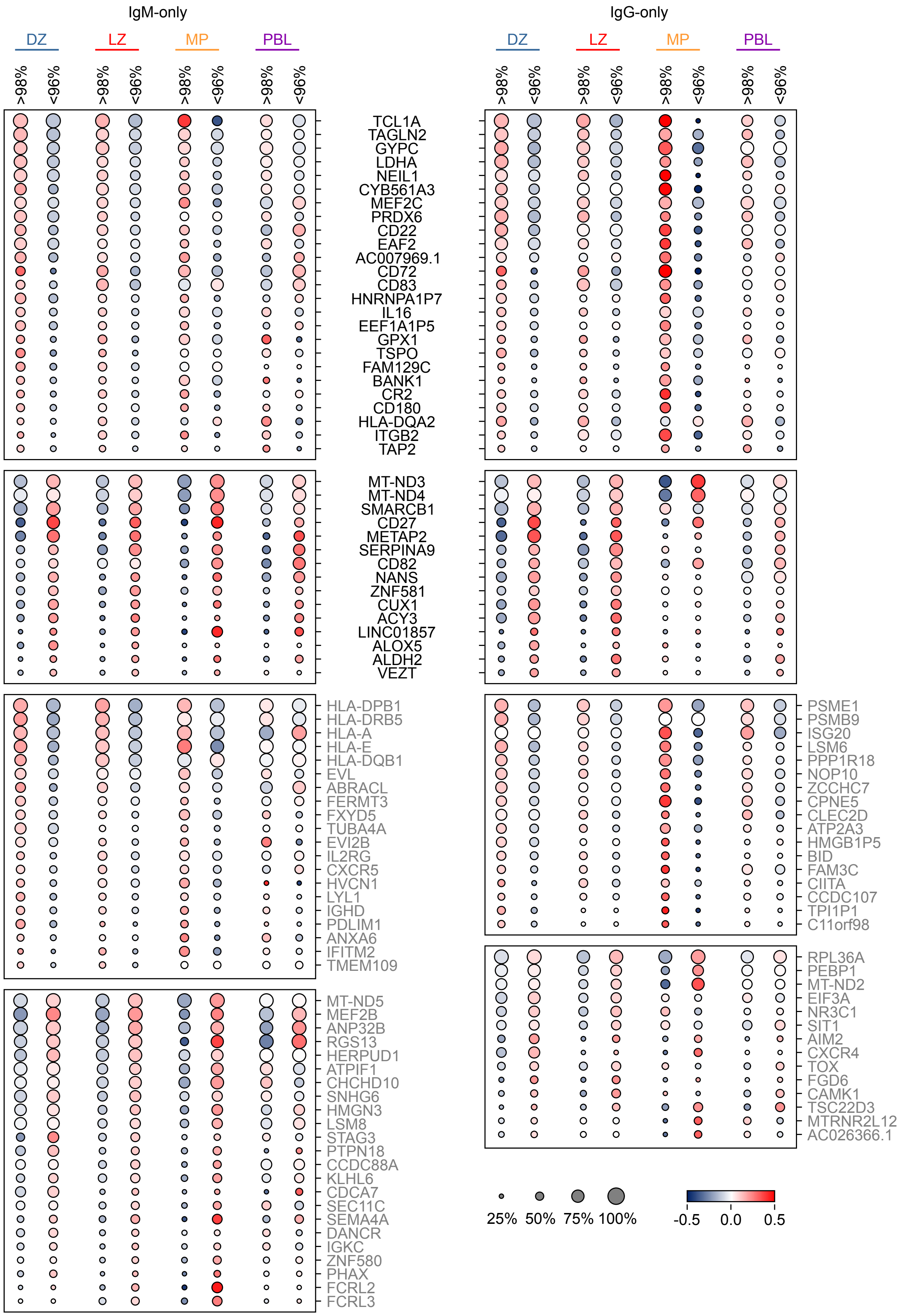

Supplementary Figure 2. Gene signatures associated with the variable-region mutational load in the major GC subgroups. Differential gene expression analysis between GC B cells with high (>98%) and low (<96%) variable (V)-region germline identity in each of the major GC subgroups (DZ, LZ, memory precursors, MP, and plasma blasts, PBL). The analysis was performed separately in cells expressing IGHM (left panel) or IGHG (right panel). Genes that were significantly differentially expressed in at least two subgroups are displayed: the upper panel (black labels) shows genes with a consistent pattern in both $IGHM^{+}$ and $IGHG^{+}$ cells, while the lower panel (gray labels) shows genes detected only in one of the analyses. The size of the dot indicates the percentage of cells with detectable expression, and the color reflects the relative gene expression shown as the z-scored average log2 normalized expression within a group.
